# New stent for transapical mitral valve replacement in acute swine experiment

**DOI:** 10.1186/s13019-021-01483-1

**Published:** 2021-04-21

**Authors:** Yu Zou, Peng Teng, Liang Ma

**Affiliations:** Department of Cardiovascular Surgery, the First Affiliated Hospital, College of Medicine, Zhejiang University, 79 Qing Chun Road, Hang Zhou, 310003 Zhejiang Province China

**Keywords:** Mitral regurgitation, Transcatheter mitral valve replacement, Self-expanding valved stent, Transapical

## Abstract

**Background:**

Many patients with mitral regurgitation are denied open-heart surgery due to perceived high risk. Transcatheter mitral valve replacement is a therapeutic alternative for patients at high surgical risk. This study aimed to assess the feasibility of a new self-expanding valved stent for transcatheter mitral valve replacement via apex in an acute animal model.

**Methods:**

Eight porcine experiments were performed in the acute study. A left thoracotomy was performed, and the new self-expanding transcatheter valved stent was deployed under fluoroscopic guidance in the native mitral annulus via apex. Hemodynamic data were recorded before and after implantation. Mitral annulus diameter and valve area were measured using echocardiography. Transvalvular and left ventricular outflow tract pressure gradients were measured using invasive methods.

**Results:**

Seven animals underwent successful transapical mitral valve replacement; the implantation was unsuccessful in one animal. The mean procedure time, defined from placement to tightening of the purse-string suture, was 17.14 ± 7.86 min. Hemodynamic data before and after transapical mitral valve replacement showed no difference in statistical analysis. The mean diameter of the self-expanding device after implantation was 2.58 ± 1.04 cm; the mean functional area was 2.70 ± 0.26 cm^2^. Trace-to-mild central and paravalvular leaks were detected in 7 valves. The mean pressure gradient across the self-expanding device was 2.00 ± 0.82 mmHg; the corresponding gradient across the LVOT was 3.28 ± 1.11 mmHg. Postmortem evaluation confirmed precise device positioning in 7 animals with no signs of LVOT obstruction.

**Conclusion:**

Transcatheter mitral replacement of the new valved stent was confirmed feasible in acute preclinical models. The new stent reveals optimal design parameters.

## Introduction

Mitral regurgitation is one of the most common heart valve diseases worldwide [1, 2]. Although valve repair is the treatment of choice [3, 4], replacement is still the gold standard, especially for complex cases or repair failure [5]. However, many patients are denied open-heart surgery due to perceived high risk [6, 7].

In the past decade, transcatheter aortic valve implantation (TAVI) is used increasingly with exciting results as a surgical alternative for high-risk patients [8, 9]. Transcatheter mitral valve replacement (TMVR) is currently in the early clinical stage of development with reports of several devices and delivery systems [10, 11]. Although the results have been encouraging, the technique still faces many challenges due to the complex anatomy of the mitral apparatus, high ventricular pressure, and mitral annulus motion [12].

We developed a new self-expanding valved stent for transcatheter mitral valve replacement via apex. This study tests its feasibility in an acute animal model.

## Methods

### The new valved stent

**Fig. 1:**
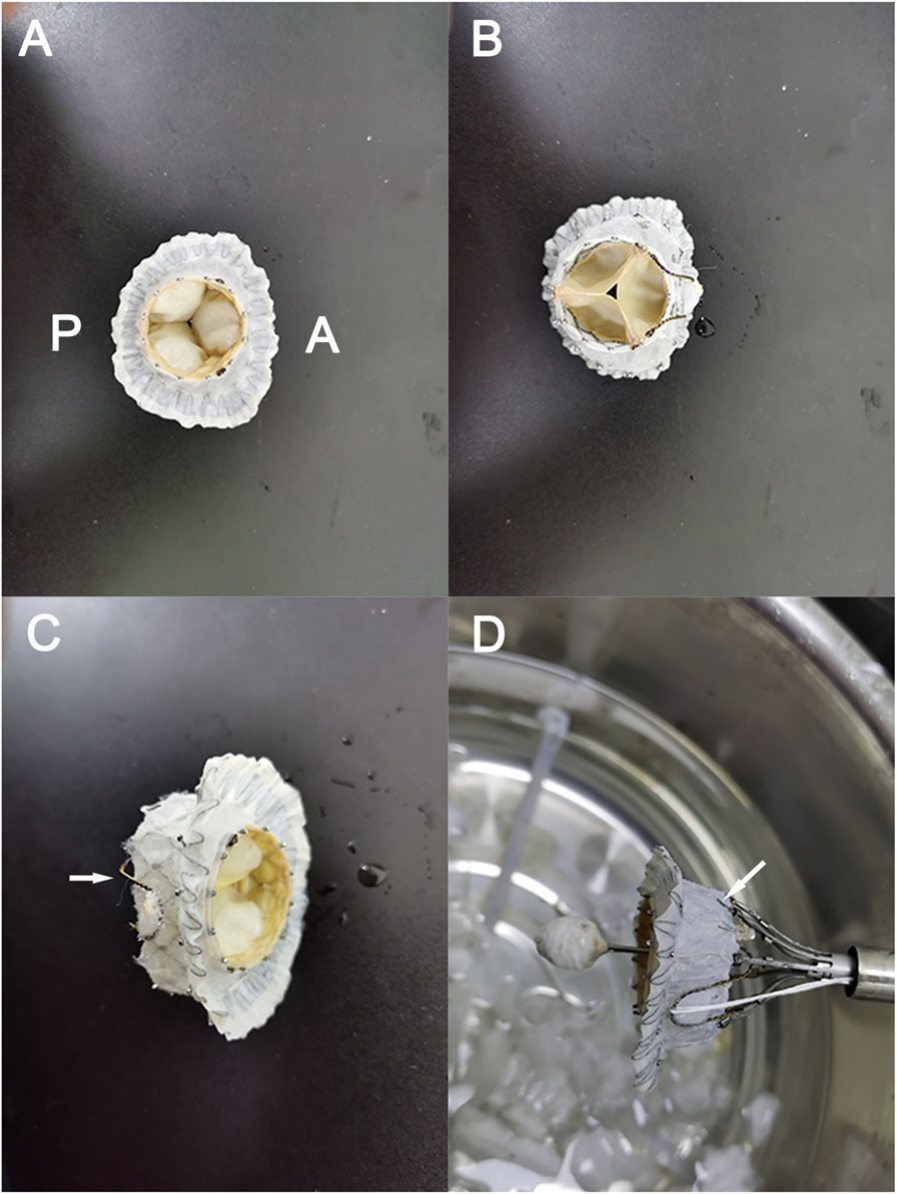
The newly designed self-expanding nitinol valved stent. **a** Atrial view (A: facing anterior leaflet. P: facing posterior leaflet). **b** Ventricular view. **c** Side view. **d** The valved stent loaded into the delivery system. The clip (**c** and **d**) on the ventricular portion holds the anterior leaflet of the mitral valve

The new stent (Fig. 1) is a self-expandable nitinol stent consisting of two components: (1) atrial fixation system with a D-shaped design; (2) circular ventricular part accommodating a trileaflet valve made of bovine pericardium. The D-shaped atrial frame that engages with the native mitral annulus helps stent fixation and reduces paravalvular leak. The flange sections of the two edges are wider than the flat aspect and the arc, specifically designed to fit the saddle-shaped mitral annulus. The asymmetric flange reduces the impact on the left ventricular outflow tract and prevents stent displacement. The trileaflet bovine valve is mounted on the circular ventricular body. A clip on the ventricular portion corresponds to the flat aspect of the D-shaped stent (Fig. 1c). The anterior leaflet is held between the clip and the ventricular body. A few struts are arranged radially on the ventricular component for anchoring (Fig. 1d arrow). Three tethering strings attached to the lower part of the stent are fixed to the apex of the heart to prevent stent displacement. The stent frame is covered by a polyester fabric skirt to minimize paraprosthetic leaks, facilitating tissue ingrowth for long-term fixation and sealing.

### Delivery system

The delivery system (Fig. 1d) is designed for transapical access only. The prosthesis is compressed and loaded into a 33Fr delivery capsule. It is advanced into the left ventricle via apex over the wire. Three knobs of the delivery system correspond to the three stages of deployment: atrial body expanding, ventricular expanding, and clip releasing. Rapid ventricular pacing is not required during the entire procedure.

### Animal preparation

The study was approved by the local Ethics Research Board. Animals received care in compliance with the “Guide for the Care and Use of Laboratory Animals” prepared by the Institute of Laboratory Animal Resources and published by the National Institute of Health (NIH publication 85–23, revised 1985).

Eight porcine experiments (mean body weight of pigs was 46.45 ± 3.72 kg) were performed in this acute study. After administering general anesthesia with tracheal intubation and mechanical ventilation (intramuscular ketamine 22 mg/kg and atropine 0.8 mg/kg, intravenous thiopental 15 mg/kg for induction, and isoflurane 2.5% for maintenance anesthesia), a coronary sinus electrode was inserted into the right internal regular vein using digital subtraction angiography (DSA) guidance to confirm the position of the mitral annulus. The right femoral artery was catheterized for monitoring blood pressure and blood sampling. The left femoral artery was implanted with a 5F pigtail into the aortic root for aortic valve localization and angiograph. Arterial pressure, central venous pressure, oxygen saturation, and electrocardiography were monitored continuously.

### Transapical procedure

**Fig. 2:**
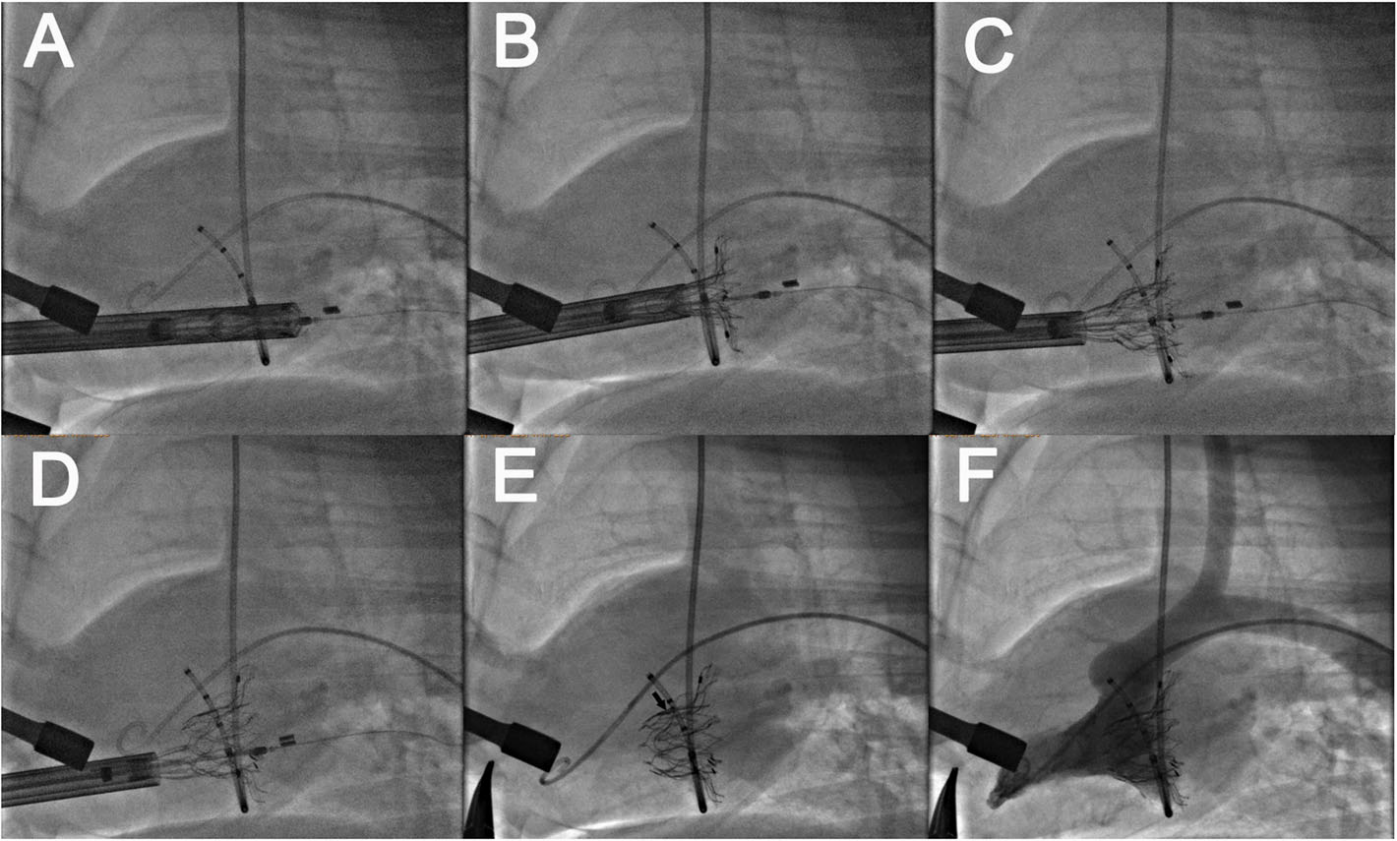
**a** Delivery system advanced into the left atrium. **b** Atrial brim expansion. **c** Ventricular body deployed. **d** The clip released, and the stent separated from the conveyor completely. **e** The delivery system was withdrawn, and the stent was working. The arrow shows the clip. **f** Left ventricular angiogram shows no paravalvular regurgitation

A 5-cm left thoracotomy was performed. Double-purse string sutures were placed at the optimal access site of the left ventricular apex. The valved stent was 10% larger than the diameter of the native mitral annulus measured by CT and epicardial echocardiography. Left ventricular angiography was performed to determine the position of the mitral annulus. A stiff wire was introduced across the mitral valve into the pulmonary vein; the delivery system was advanced along the guide wire into the left atrium, using DSA guidance. The atrial brim was expanded first. It was necessary to gradually adjust the conveyor to the proper position so that the flat aspect of the D-shaped stent was aligned with the aorta. (There is a directional mark on the conveyor). The delivery system was pulled down until the atrial flange was seated firmly on the floor of the left atrium. The ventricular body was deployed without rapid ventricular pacing, and the clip was released to hold the anterior valve. After achieving stability, the stent was completely separated from the conveyor. Three tethering strings attached to the stent were pulled out of the apex with the conveyer withdrawn. The length of the tethering strings was adjusted under real-time monitoring using echocardiography. The apical access site was closed with purse-string sutures. The tethering strings were fixed at the epicardial surface by knotting the polyester felts. The procedure was performed under fluoroscopic guidance (Fig. 2).

**Fig. 3:**
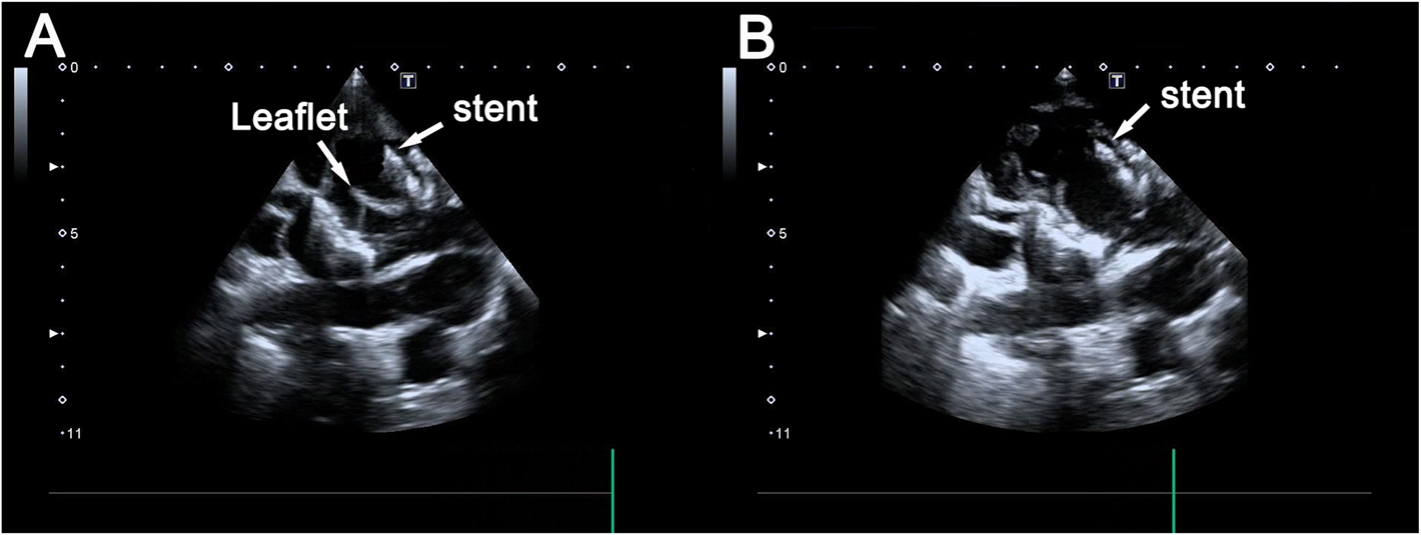
Epicardial echocardiography reveals excellent device function with complete leaflet closing (**a**) and opening (**b**)

Device function and hemodynamic impact were assessed using epicardial echocardiography (Fig. 3) and angiography (Fig. 2f) at baseline and 30 min after implantation. The transprosthetic and trans-LVOT pressure gradients were measured. Left ventricular angiography was performed.

**Fig. 4:**
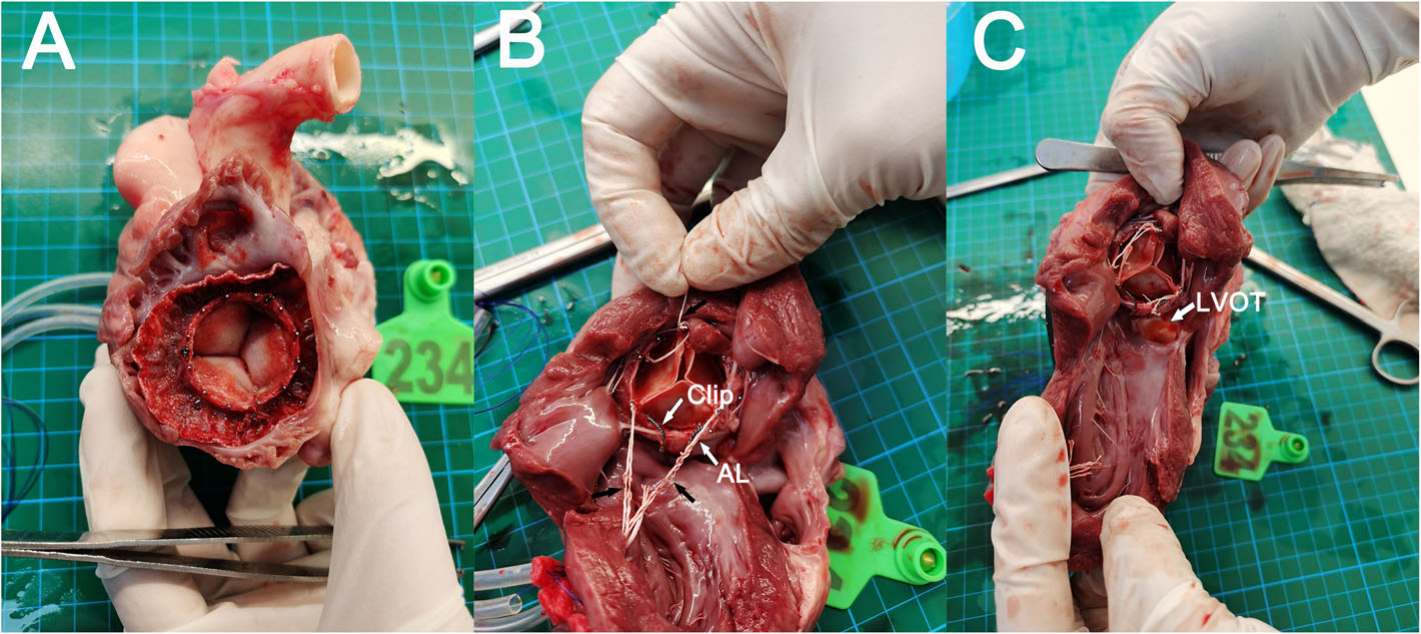
Postmortem evaluation reveals accurate placement. **a** Left atrial side. **b** Left ventricular side (AL: anterior leaflet). Three tethering strings (black arrow) are attached to the lower part of the stent. **c** No signs of LVOT obstruction

Continuous hemodynamic measurements were recorded for another 6–8 h. The animals were sacrificed for postmortem evaluation and device inspection (Fig. 4).

### Statistical analysis

Data were analyzed using SPSS v19 software for Windows. Variables were reported as mean ± standard deviation (SD), and Student’s T-test was used for comparison.

## Results

Seven of eight animals underwent successful transapical mitral valve replacement. In one animal, device positioning failed because of premature stent separation from the delivery system due to incorrect placement, which led the ventricular portion of the stent to migrate into the left atrium. The mean procedure time, defined from placement to tightening of the purse-string suture, was 17.14 ± 7.86 min. Hemodynamic data before and after transapical mitral valve replacement are shown in Table 1. The mean diameter of the native mitral annulus was 2.46 ± 1.00 cm, and the mean mitral valve area was 4.58 ± 0.29 cm^2^ on echocardiographic evaluation; the mean diameter of the self-expanding device after implantation was 2.58 ± 1.04 cm, and the mean functional area was 2.70 ± 0.26 cm^2^. No statistical difference existed in mitral diameter before and after implantation. However, the functional mitral valve areas showed significant differences before and after the procedure (Sig = 0.00 , *p* < 0.05). Of the 7 successful implants, all valve leaflets had normal mobility and function. Trace or mild central and paravalvular regurgitation was detected in 7 valves (Table 2). The mean pressure gradient across the self-expanding device was 2.00 ± 0.82 mmHg; the corresponding gradient across the LVOT was 3.28 ± 1.11 mmHg (Table 1).

**Table 1:** Procedure and hemodynamic data

No.	Weight (Kg)	HR (bpm)		BP (mm Hg)		CVP (mm Hg)		Valve diameter (mm)		Valve area^a^ (cm^2^)		Transapical time (min)	Across valve (mm Hg)	Across LVOT (mm Hg)
		Pr	Po	Pr	Po	Pr	Po	Native	stent	Native	Stent			
1	43	125	130	123/92	121/70	10	9	23	25	4.30	2.53	33	3	5
2	47	120	126	110/80	100/67	8	7	25	27	4.26	2.68	20	2	3
3	53.6	112	120	103/62	106/72	9	7	25.5	27	5.00	3.20	18	2	4
4	50	123	112	98/53	101/58	11	8	25.1	27	4.23	2.83	11	1	4
5	45.3	130	121	107/70	N	8	N	23.2	25	4.12	N	failed	n.a.	n.a.
6	43.7	110	113	117/73	120/70	10	8	23.5	25	4.31	2.38	10	1	2
7	46	126	111	112/68	118/72	12	10	24	25	4.50	2.63	13	2	3
8	43	112	130	120/81	118/73	7	9	23.2	25	4.18	2.67	15	3	2
Mean	46.45	119	120	111/72	112/69	9.38	8.29	24.06	25.75	4.36	2.70	17.14	2.00	3.28
SD	3.72	7.54	7.84	8.55/12.11	9.29/5.18	1.69	1.11	1.00	1.04	0.26	0.26	7.86	0.82	1.11

*Across LVOT* Gradient across LVOT, *Across valve* Gradient across valve, *BP* Blood pressure, *CVP* Central venous pressure, *HR* Heart rate, *n.a.* Not available (due to failed implantation), *post* after implantation, *pre* before implantation, *SD* Standard deviation

^a^Differences between native and stent were statistically significant

**Table 2:** Mitral regurgitation

No.	CL	PVL
1	Mild	Mild
2	None	Mild
3	trace	mild
4	Mild	Trace
5	None	Moderate to Severe
6	Mild	Mild
7	Trace	mild
8	Trace	Mild

*CL* Central leak, *PVL* Perivalvular leak

Postmortem evaluation confirmed precise device positioning in 7 animals, with no LVOT obstruction (Fig. 4d). For the failed case, the ventricular body of the stent facing the posterior annulus was deployed at the left atrium. The body facing the anterior annulus was at the true position, securing the anterior leaflet between the clip and the stent. LVOT was not hindered.

## Discussion

Unlike transcatheter aortic valve replacement (TAVR), the development of transcatheter mitral valve replacement faces unique challenges. The mitral valve is a complicated anatomic apparatus, including mitral leaflets, chordae, and papillary muscles. The non-circular saddle-shaped annulus is pliable and changes during the cardiac cycle. Several vital structures, such as the circumflex coronary artery, atrioventricular node, and left ventricular outflow tract, are adjacent to the mitral valve [13]. Specifically, the anterior leaflet of the mitral valve is a part of the left ventricular output tract [14]. All of these features create a series of technical difficulties.

Although several challenges exist in TMVR, the TMVR system is rapidly expanding, with over 10 devices currently under development [15]. Several devices, such as Tendyne™ (Abbott Vascular, Santa Clara, United States), Intrepid (Medtronic, MN, United States), Tiara® (NeoVasc, Richmond, Canada), CardiaQ (Edwards Lifesciences, Irvine, United States), are presently under clinical evaluation [16–20]. The transapical approach is the chief approach for TMVR. It provides easy access to the mitral valve and a simple stent-release procedure. The use of large-bore catheters (34- to 36-F) is allowed in the transapical approach. The transseptal approach has emerged as a hopeful alternative for TMVR. The technical difficulty and smaller sheath size limit its development [21].

The atrial portion of our device is “D” shaped to match the native mitral annulus. It is conducive to stent fixation and reduces paravalvular leakage. The flange section of the two edges is wider than the flat aspect and the arc, specifically designed to fit the saddle-shaped mitral annulus. The asymmetric flange minimizes left ventricular outflow tract obstruction and prevents stent displacement. A circular ventricular stent has three anchoring structures. First, radially struts are arranged on the ventricular component of the valved stent to penetrate the mitral leaflet and the valvular apparatus and prevent the device retrograde dislodgement into the atrium during systole. Second, a clip on the ventricular portion corresponds to the position of the anterior leaflet of the mitral valve. It is released to hold the anterior leaflet of the mitral valve after the ventricular component opens completely. The design minimizes left ventricular outflow tract obstruction caused by the native anterior leaflet. It also prevents the movement of the device into the left atrium. During the operation, we did not deliberately capture the anterior leaflet. As long as the flat aspect of the D-shaped stent is aligned with the anterior annulus of the mitral valve, the clip can easily hold the anterior leaflet after the left ventricular body is fully opened. Third, three tethering strings are attached to the lower part of the stent and fixed to the apex of the heart to prevent stent displacement. The early experiments showed that the posterior side of the stent shifted to the left atrium during systole. This finding may be related to the insufficient anchoring force between the device and the posterior annulus. We did not include a clip on the posterior of the stent as Tiara to avoid the risk of penetrating the posterior wall of the left ventricle. Therefore, we added three strings attached to the stent symmetrically that could be pulled out with the delivery system and sutured to the apex. Current TMVR devices presenting anchoring systems with clips or tethering strings have proved to be reliable. Tiara® has three clips, two anterior and one posterior. Medtronic has two clips, called support arms. In our device, the single-clip design makes it easier to hold the anterior leaflet. Tendyne™ uses a ventricular fixation system connected to the LV apex through a polyethylene tether [10]; the complementary combination of the clip and tether string achieves good results. No displacement happened in the seven animals.

Our self-expanding stent valve achieved good hemodynamic performance in the acute porcine model with no stent displacement and left ventricular outflow tract obstruction. Although a significant difference exists in the effective functional area between the stent valve and the natural valve, it was comparable to the conventional bioprosthetic valve. Therefore, this study confirmed the feasibility of the new valved stent and the delivery system. We plan to conduct a long-term animal experiment to observe the reliability of valve stents.

A limitation of this study is the use of healthy animal models with normal cardiac anatomy and physiology. The healthy pig is significantly different from the patient presenting with functional mitral regurgitation. Another limitation is that the study is an acute experiment. It is necessary to carry out chronic experiments to verify the reliability of valve stents.

## Conclusions

The new transapical valved stent was feasible and safe for mitral valve replacement and showed promising hemodynamic performance in an acute study.

## Data Availability

All data generated or analyzed during this study are included in this published article.

## Abbreviations

LVOT: Left ventricular outflow tract
TAVI: Transcatheter aortic valve implantation
TMVR: Transcatheter mitral valve replacement
TAVR: Transcatheter aortic valve replacement
